# Visual target distance, but not visual cursor path length produces shifts in motor behavior

**DOI:** 10.3389/fpsyg.2014.00225

**Published:** 2014-03-17

**Authors:** Nike Wendker, Oliver S. Sack, Christine Sutter

**Affiliations:** Department of Work and Cognitive Psychology, RWTH Aachen UniversityAachen, Germany

**Keywords:** aftereffect, tool use, dimensional overlap, contextual interference, human information processing

## Abstract

When using tools effects in body space and distant space often do not correspond. Findings so far demonstrated that in this case visual feedback has more impact on action control than proprioceptive feedback. The present study varies the dimensional overlap between visual and proprioceptive action effects and investigates its impact on aftereffects in motor responses. In two experiments participants perform linear hand movements on a covered digitizer tablet to produce ∩-shaped cursor trajectories on the display. The shape of hand motion and cursor motion (linear vs. curved) is dissimilar and therefore does not overlap. In one condition the length of hand amplitude and visual target distance is similar and constant while the length of the cursor path is dissimilar and varies. In another condition the length of the hand amplitude varies while the lengths of visual target distance (similar or dissimilar) and cursor path (dissimilar) are constant. First, we found that aftereffects depended on the relation between hand path length and visual target distance, and not on the relation between hand and cursor path length. Second, increasing contextual interference did not reveal larger aftereffects. Finally, data exploration demonstrated a considerable benefit from gain repetitions across trials when compared to gain switches. In conclusion, dimensional overlap between visual and proprioceptive action effects modulates human information processing in visually controlled actions. However, adjustment of the internal model seems to occur very fast for this kind of simple linear transformation, so that the impact of prior visual feedback is fleeting.

## Introduction

Humans use tools to either extend their own capacities, to enlarge and strengthen single parts of their body, or as a way to sort out problems. In modern live we are confronted with technologies that transform body movements into tool movements by linear and dynamical perturbations (e.g., a computer mouse), and/or by inverting movement directions (e.g., a laparoscope in minimal-invasive surgery). These sensorimotor transformations challenge the human information processing system, since the sensory feedback from the moving hand (proximal action effect) and the sensory feedback from the moving effective part of the tool (distal action effect) do not correspond.

For controlling human actions, it is widely accepted that the proximal movement-effect loop is essential to generate an action plan from the very beginning. This so-called ideo-motor principle of action planning holds that agents select, initiate and execute a movement by activating the anticipation of the sensory codes of the movement's effects (James, 1890; Greenwald, 1970; for an overview see Hommel et al., 2001). However, in tool use distal action effects predominate action control while proximal action effect are attenuated or even ignored (Mechsner et al., 2001; Sutter and Ladwig, 2012; Wang et al., 2012; Ladwig et al., 2013; for an overview and limits in distal action effect control see, e.g., Sutter et al., 2013).

Fourneret and Jeannerod (1998) demonstrated that participants are not very aware about their own hand movements. Participants traced sagittal lines on a graphic tablet using a stylus held in their right hand while a mirror hid their hand movements. The mirror presented visual feedback, so that participants saw their lines projected from a computer screen. While in control trials the line was exactly the same as seen in the mirror, in perturbed trials the line appeared to deviate in one direction “right or left” by a variable angle (2, 5, 7, or 10°). The main finding was that participants consistently displaced their hand in the opposite direction for drawing a visually sagittal line. When participants were asked in which direction they thought their hand had moved, participants largely underestimated their hand deviation in perturbed trials.

**Figure 1:**
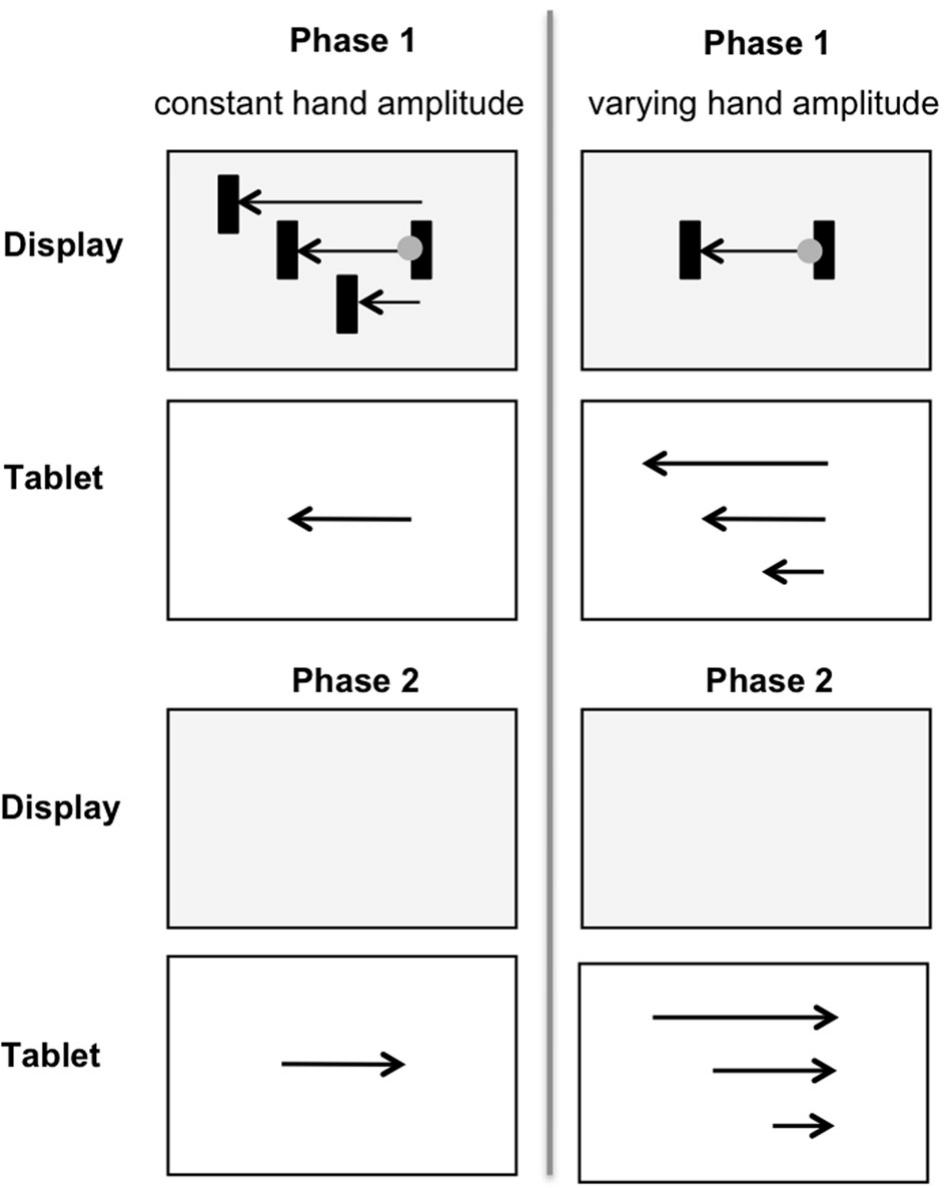
**In phase 1 (upper part) cursor amplitudes were shorter, equal to, or longer than the constant hand amplitude (left) or cursor amplitudes remained constant across trials, while the hand amplitude was shorter, equal to, or longer (right)**. In phase 2 (lower part) the initially performed hand amplitude was replicated without any visual feedback.

Ladwig et al. (2012) investigated the recall of proprioceptive information after performing a hand movement with perturbed visual feedback. In phase 1 (Figure 1, upper part), participants were asked to move the cursor horizontally from one target bar to the other by moving a pen on a digitizer tablet. The cursor amplitude presented on the display was shorter, equal to or longer than the hand amplitude. The digitizer tablet and the hand were covered with an occluder, so that participants only received perturbed visual feedback on the display. After reaching the target area the movement direction had to be reversed. In phase 2 (Figure 1, lower part) participants were asked to replicate the formerly performed hand amplitude as accurately as possible without any visual feedback. In one condition the hand amplitude was held constant while the cursor amplitudes on the display were shorter or longer than the hand amplitudes (Figure 1, left). In another condition the cursor amplitude was constant and hand amplitudes were shorter or longer (Figure 1, right). In control trials in each condition hand and cursor amplitudes were equal.

In untransformed trials participants replicated movements very accurately. In perturbed trials hand amplitudes prominently shifted, influenced by the formerly received visual feedback. When participants had seen shorter (longer) cursor amplitudes the replicated hand amplitudes were accordingly shorter (longer). These shifts occurred in constant and varying hand amplitudes, but they were more pronounced when proximal effects varied. That means visual information from phase 1 biased motor replications in phase 2. The authors interpreted the shifts as a visual aftereffect. Common coding approaches (e.g., Prinz, 1997; Hommel et al., 2001) propose that sensory information from perceived actions and intended actions are coded and stored in a common representational domain. As a result of this, sensory information from different senses is likely to interact and to affect subsequent action control. The findings by Ladwig et al. (2012) demonstrate this kind of cross talk in terms of visual aftereffects. Because, if visual information from phase 1 could have been completely ignored in motor replication (phase 2), then inaccuracy in motor replications should have been independent from the visual information in phase 1. But this was not the case. Ladwig et al. (2012, 2013) observed a systematic pattern of under- and overshoots that depended on the length of the formerly seen cursor amplitudes: When participants had seen shorter (longer) cursor amplitudes (phase 1) the replicated hand amplitudes in phase 2 were accordingly shorter (longer). This pattern was even observed for constant hand amplitudes but varying cursor amplitudes. In this condition, replicated hand movements could have been performed without any corrections of the previously-used motor program (Wolpert and Flanagan, 2001). That motor replications were still influenced from the formerly perceived visual information speaks in favor of the common representational domain of sensory information from perceived actions and intended actions (e.g., Prinz, 1997; Hommel et al., 2001).

Furthermore, the theory of event coding and the dimensional overlap model (Kornblum et al., 1990; Kornblum and Lee, 1995; Hommel et al., 2001) assume that when perceptual stimuli share some features with planned actions, these stimuli can either foster those actions or interfere with them depending on their similarity. Dimensional overlap is treated as a dichotomous variable and describes the match or mismatch between stimulus (S) and response (R) along functionally separable object dimensions (Kornblum and Lee, 1995). Orientation, size and shape are object dimension, whereas “vertical,” “long,” or “curved” are features on those dimensions (Treisman and Gelade, 1980). The impact of the dimensional overlap on aftereffects was investigated in a second condition (Ladwig et al., 2012). The horizontal hand motion on the tablet produced a vertical cursor motion on the display. The orientation of hand and cursor motion did not longer overlap (horizontal vs. vertical), and this resulted in smaller aftereffects when compared to the condition in which the orientation of hand and cursor motion did overlap (both horizontal).

The aim of the present study is to further investigate the impact of dimensional overlap on aftereffects in motor replications. For this we adapt the task introduced by Ladwig et al. (2012). Again, participants move the cursor on the display from a start position to a target, but now the cursor motion follows the shape of an inverted U while the hand motion still follows a straight horizontal line (Figure 3, upper part). In the condition *perturbed cursor motion* (Figure 3, left) the length between start and target area (= visual target distance) and the length of the hand motion are similar and remain constant. The variable length (short, middle, long) and the shape of the cursor trajectory (∩-shaped) are dissimilar from the horizontal hand motion. When features are similar (dissimilar), then dimensions do (not) overlap. In the condition *perturbed hand motion* (Figure 3, right) the constant visual target distance and the variable length of the hand motion are similar (middle) or dissimilar (short, long). The constant length and shape of the cursor trajectory are dissimilar from the varying horizontal hand motion. In phase 2 participants replicate the formerly performed hand amplitude (Figure 3, lower part).

The variation of length and shape of hand and cursor motion decouples two different relations in visually controlled aiming movements: First, the relation between hand motion and cursor motion (dissimilar length and dissimilar shape). And second the relation between hand motion and visual target distance (similar or dissimilar length and similar shape). The experimental variations of the dimensions shape and length, and their dimensional overlap in phases 1 and 2 are depicted in Table 1.

**Table 1:**
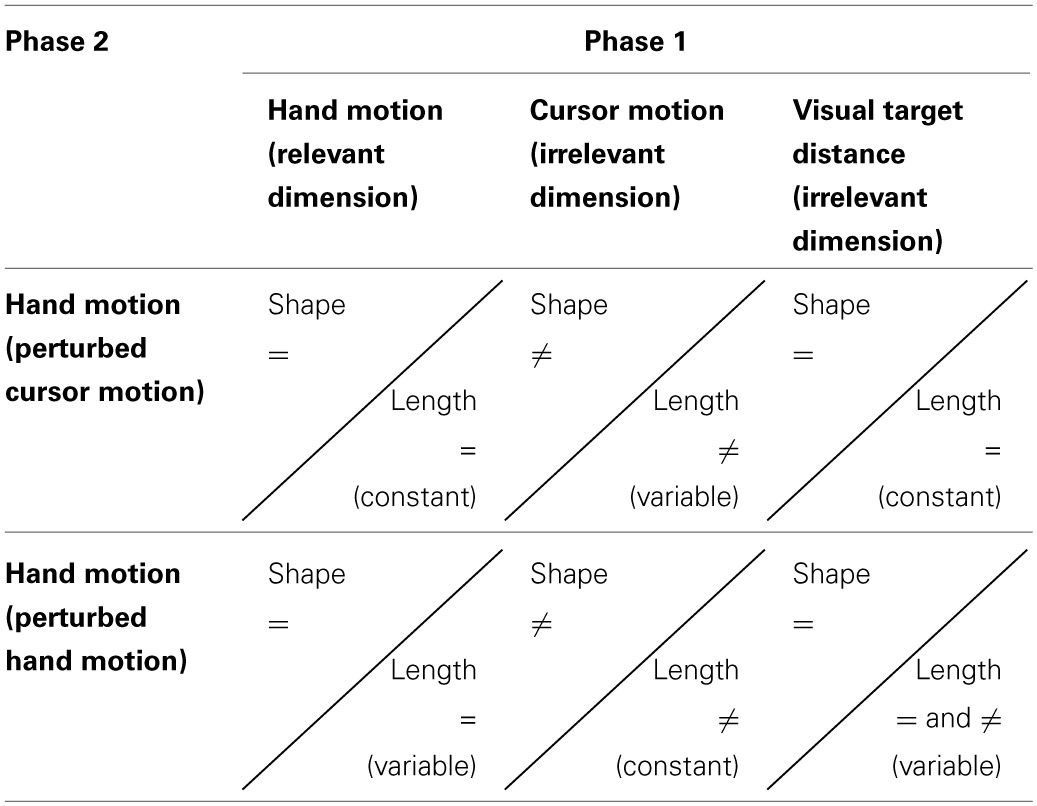
**The experimental variations of the dimensions shape and length, and their dimensional overlap in phases 1 and 2 (“=”, similar; i.e., dimension does overlap; “≠”, dissimilar; i.e., dimension does not overlap)**.

Thus, the first hypothesis (H1) concerns the dimensional overlap and its impact on aftereffects. H1a: If it is the relation between hand motion and cursor motion (dimensions do not overlap) that accounts for the aftereffects, then in both conditions aftereffects should be present in terms of overshoots, since the cursor motion is always longer than the hand motion. Overshoots in motor replications should increase from short to long cursor motions. H1b: However, if it is the relation between hand amplitude and visual target distance (dimensions do overlap), then we do not expect any aftereffects in the condition with perturbed cursor motions. In the condition with perturbed hand motions (dimensions do or do not overlap) aftereffects should follow the same pattern as observed by Ladwig et al. (2012). When the visual target distance is shorter (longer) than the hand amplitude, participants should undershoot (overshoot). Therefore, we do not expect any aftereffects when the relation is 1:1. The ideo-motor principle (James, 1890; Greenwald, 1970) would predict the same pattern of results as H1b. Actions are cognitively represented with respect to the goal of the action, not with respect to the way we achieve the action's goal. In this sense, the relation between hand amplitude and visual target distance (H1b) should be more important for controlling actions than the relation between hand amplitude and cursor path length (H1a).

The second hypothesis (H2) considers the impact of the context in phase 1 on aftereffects in motor replications (phase 2). The contextual interference effect (Magill and Hall, 1990; Guadagnoli and Lee, 2004) describes a benefit for (motor) skill acquisition when tasks are presented in blocked practice condition, but a disadvantage on retention and transfer, and the other way around for the random practice condition. The reason for this seems to be due to the simple and automated (learning a task in one context—blocked practice blocks) vs. elaborated (learning a task in multiple contexts—random practice blocks) cognitive processing when learning a task. In our experiments, the task irrelevant visual feedback in phase 1 can be considered as the context in which the motor task is performed. In Experiment 1 we present two small, randomized blocks of trials in which three different gains perturb either the cursor motion (one block: cursor motion varying, hand motion constant) or the hand motion (another block: hand motion varying, cursor motion constant). In Experiment 2 we present the same trials of perturbed cursor or hand motions as in Experiment 1 but randomly mixed within a block. We assume that participants in Experiment 1 may, at least implicitly realize that one aspect of the task in phase 1 remains constant within a block, either the hand motion or the cursor motion. The motion constancy and the smaller set size of contexts to be learned in Experiment 1 should lead to smaller aftereffects when compared to Experiment 2 in either both conditions (H2a) or in the condition with perturbed hand motions only (H2b).

Finally, we explore the following research question how experience shapes subsequent motor behavior. Prior reaching a familiarized visual target reduced subsequent reaching variability for this target position, but also reduced subsequent reaching accuracy for other target positions (Verstynen and Sabes, 2011). In other words, performance in target repetitions is better than in target switches. This makes perfect sense. Movements are usually pre-programmed with the previously-used internal model (Wolpert and Flanagan, 2001). When sudden changes (e.g., a gain change) occur, the motor system compensates for and adapts to these changes by modifying the pre-programmed action during movement execution (e.g., Rieger et al., 2005). Consequently, any error at that time reflects the specification of the pre-programmed movement. In the present experiment, participants did not receive any visual feedback in phase 2. Thus, they were not able to observe the difference between the to-be-replicated hand amplitude and their actual replication. However, this is relevant information for the motor system to adjust the forward model (Wolpert and Flanagan, 2001). Thus, in the present experiment the forward model can't be adjusted if the gain changes from trial to trial (switch condition). However, if it is repeated, then the repeated closed-loop control in phase 1 function in a way to adjust the internal model. Consequently, the forward model becomes more accurate and smaller aftereffects are expected for gain repetitions than for gain switches.

## Experiment 1

### Methods

#### Apparatus, task, and stimuli

**Figure 2:**
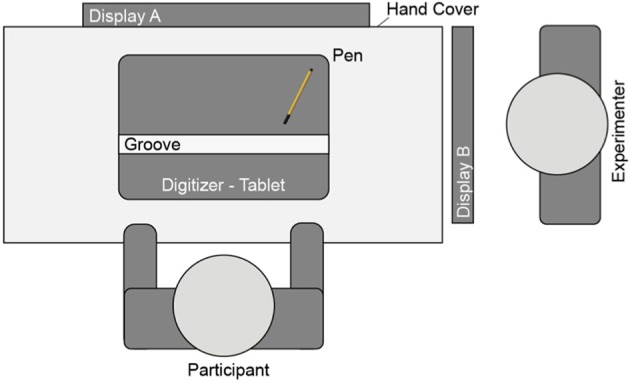
**Experimental setting**.

The experimental setting (Figure 2) was the same as used by Ladwig et al. (2012). Participants sat in a dimly lit room in front of a DIN-A3 digitizer tablet (WACOM Intuos2, 100 Hz sampling rate). A wooden cover with a curtain prevented direct vision of the digitizer tablet and the participant's hand. In Experiment 1a the experimental tasks and cursor motions were presented on a 22” color CRT display, with a distance of approximately 58 cm between participant and display (Iiyama HM204DT, Vision Master Pr514, 100 Hz refresh rate, 1024 × 768 pixels). Moving the tip of the pen (WACOM Intuos2 Grip Pen) horizontally inside a cut out groove mounted onto the digitizer tablet (width and length of the groove: 0.4 and 50 cm) controlled the cursor on the display. The experimenter sat next to the participant and monitored the log file providing information about participant's performance on a separate display. An Apple Macintosh computer running Matlab software with the Psychophysics Toolbox extension controlled the experiment (Kleiner et al., 2007).

**Figure 3:**
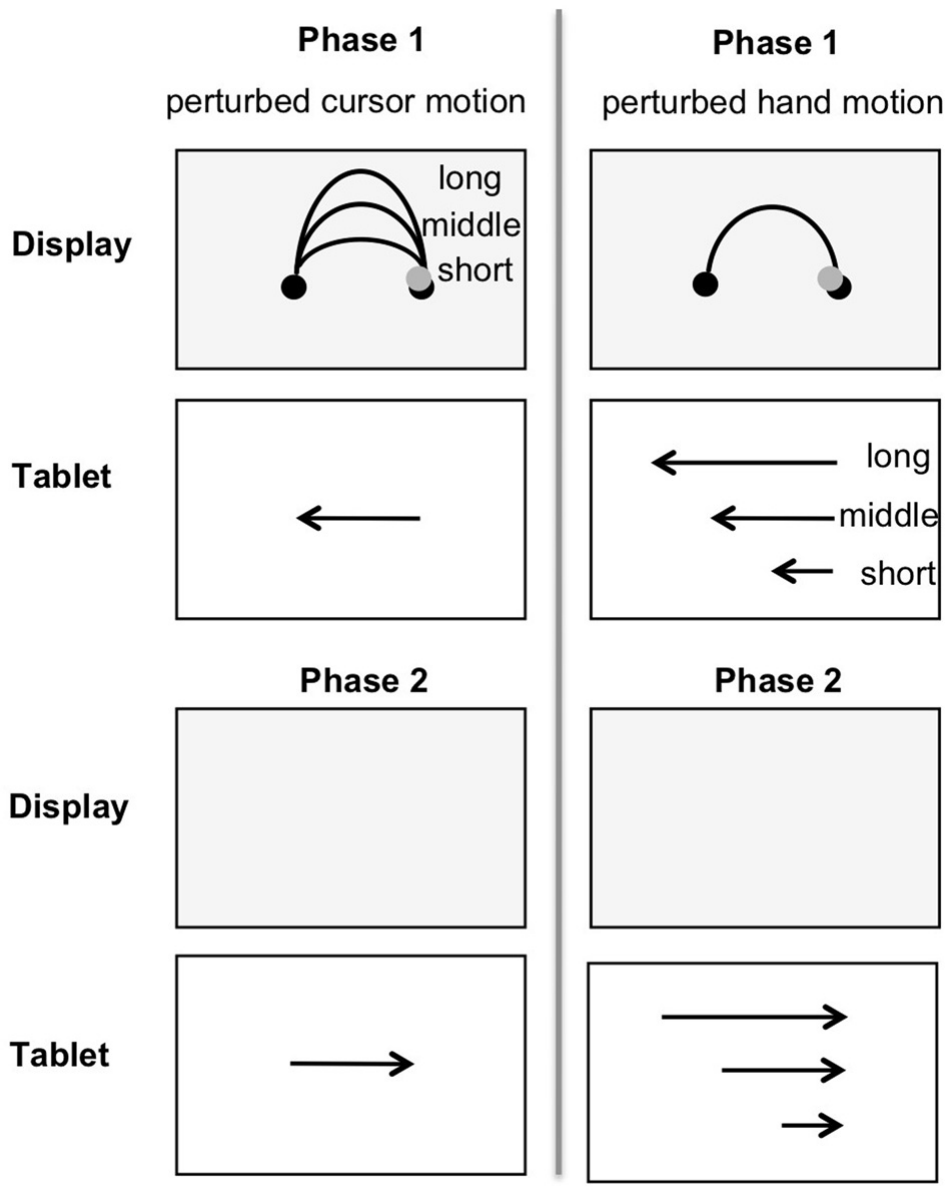
**Perturbed cursor motion (upper part, left)**. In phase 1 cursor paths were short (60 mm), middle (120 mm), or long (180 mm), while hand amplitude and visual target distance were constant (each 50 mm). **Perturbed hand motion (upper part, right)**. In phase 1 hand amplitudes were short (25 mm), middle (50 mm), or long (75 mm), while cursor path length and visual target distance remained constant (120 and 50 mm, respectively). In phase 2 (lower part) the initially performed hand amplitude was replicated without any visual feedback.

In phase 1 (Figure 3, upper part) of each trial two black dots (circle diameter 5.6 mm each, distance between dots 50 mm = visual target distance) and a gray circular cursor (circle diameter 4 mm) appeared on the white screen. The cursor was positioned onto the right dot, and the task in phase 1 required moving it to the dot on the far side as accurately as possible by moving the pen leftward along the groove on the digitizer tablet. The horizontal hand movement produced a ∩-shaped cursor motion on the display (i.e., the upper half of a vertical ellipse). When the cursor had reached the left dot, phase 2 (Figure 3, lower part) started: The screen turned blank, and participants had to move the pen back—rightward—without any visual feedback. The task in phase 2 required reproducing the initially performed hand amplitude as accurately as possible. The start position of the cursor on the left side inverted movement directions.

In phase 1 the relation between hand amplitude and cursor path length, and/or between hand amplitude and visual target distance was perturbed by three different gains. Figure 3 (left) depicts the task for *perturbed cursor motions*. The hand amplitude (*d* = 50 mm) and the visual target distance (minor axis of the ellipse) were 50 mm and remained constant across trials. The constant length of the semi-minor axis (b; Equation 1) and a varying circumference (c; Equation 2 with gain factors 0.5, 1, or 1.5) defined the ellipse. The length of the major axis was approximated (A; Equation 3). Please note, for correctly fulfilling the task the cursor motion followed only the upper half of the vertical ellipse.

(1) $$b=\frac{d}{2}=25\text{mm}$$

(2) c=gain ∗ 240mm

(3) $$A\approx \sqrt{\frac{c^{2}}{2\pi^{2}}-b^{2}}$$

For perturbed cursor motions equations 4–6 present the transformation of the x-coordinates of the pen on the tablet (*x_p_*) into visual x- and y-coordinates along the ∩-shaped cursor path (*x_c_* and *y_c_*). The length of the major axis and the circumference of the ellipse (= cursor path length) varied as a function of the applied gain. The relations between hand amplitude (50 mm) and cursor path length (60, 120, or 180 mm) were 1:0.83, 1:0.42, or 1:0.28, and the relation between hand amplitude and visual target distance (50 mm) was 1:1. In phase 2, when participants were instructed to replicate the initially performed hand amplitude, the reproduction required moving the pen by 50 mm. Thus, the motor reproduction in phase 2 required the recall of the constant motor information from phase 1, while the visual information from phase 1 was irrelevant for solving the task and had to be ignored.

(4) $$\begin{array}{l} \text{For perturbed cursor motions }\alpha =\frac{start\_x_{p}-x_{p}}{d}\;*\;180^{{}^{\circ}}\\ \text{ For perturbed hand motions }\alpha =\frac{start\_x_{p}-x_{p}}{gain}\;*\;\frac{180^{{}^{\circ}}}{d} \end{array}$$

(5) $$x_{c}=start\_x_{p}-(1-\cos(\alpha ))\;*\;b$$

(6) $$y_{c}=148\text{mm}-\sin(\alpha )\;*\;A$$

where 148 mm defines the horizontal midline of the screen, i.e., the position of the minor axis on the screen.

Figure 3 (right) depicts the task for *perturbed hand motions*. The visual target distance (minor axis of the ellipse) was again constant across trials. The constant length of the semi-minor axis (b; Equation 1) and the constant circumference (c; Equation 2 with gain factor 1) defined the ellipse. The length of the major axis was approximated (A; Equation 3). Equations 4–6 present the transformation of the x-coordinates of the pen on the tablet (*x_p_*) into visual x- and y-coordinates along the ∩-shaped cursor path (*x_c_* and *y_c_*). The elliptical cursor path remained constant and the hand amplitude varied as a function of the applied gain. The relations between hand amplitude (25, 50, or 75 mm) and cursor path length (120 mm) were 1:0.21, 1:0.42, or 1:0.63, and the relations between hand amplitude and visual target distance (50 mm) were 1:0.5, 1:1, or 1:1.5.

In phase 2, when participants were instructed to replicate the initially performed hand amplitude, the reproduction required moving the pen by 25, 50, or 75 mm. Thus, motor replications of hand amplitudes in phase 2 required the recall of varying motor information from phase 1, while the visual information from phase 1 was irrelevant for solving the task and had to be ignored.

The combination of hand amplitude (= 50 mm), cursor path length (= 120 mm) and visual target distance (= 50 mm) appeared in both conditions of perturbed cursor motions and perturbed hand motions, and were considered as control trials.

In Experiment 1b we did not provide any visual feedback on the display. Comparing results of conditions with and without feedback should clarify the impact of visual feedback on observed deviations. If visual feedback in phase 1 induced deviations in phase 2, then the hypothesized pattern of over- and undershoots should occur in Experiment 1a, but not in Experiment 1b.

A second experimenter sat opposite the participant. A perforated plastic plate (size 255 × 255 mm) was attached to the experimenter's side of the cut out groove. Two plastic blocks (95 × 15 × 9 mm) adjusted to the plate functioned as barriers and restricted the distance of the hand movement. All other materials were the same as in Experiment 1a.

In phase 1 of each trial a second experimenter adjusted both plastic barriers on the plate 25, 50, or 75 mm apart. The participant moved the pen along the groove from the right barrier to the left barrier. After movement initiation the experimenter removed the right barrier. When the pen had reached the left barrier, phase 2 started. Participants had to move the pen rightward to reproduce the initially performed hand amplitude of 25, 50, or 75 mm as accurately as possible. The start position of the pen on the left side inverted movement directions.

#### Procedure and design

Experiment 1a consisted of two blocks: In block 1 the path length of cursor motions varied [short (60 mm) vs. middle (120 mm) vs. long (180 mm)]. Cursor motions were always longer than the constant hand motions (50 mm). In block 2 the path length of hand motions varied [short (25 mm) vs. middle (50 mm) vs. long (75 mm)]. Hand motions were always shorter than the constant cursor motions (120 mm). The order of blocks was counterbalanced across participants. Participants were randomly assigned to movement directions.

Each block consisted of 45 trials (three gains with 15 repetitions each, randomly presented) and another six trials presented in advance of each block in order to familiarize subjects with the task (the same three gains as used in the experimental trials with two repetitions each, randomly assigned).

Before a block started, participants were instructed to move as accurately as possible and to produce continuous and smooth forth and back movements with the pen without interrupting. They were further instructed to reproduce the initially performed hand amplitude in phase 2 as accurately as possible and to monitor their hand motion in phase 1 carefully. At the beginning of each trial, the cursor as well as the start and target dot were presented on the screen. Participants were free to choose a start position within the groove on the tablet. That means, hand and cursor motions were not spatially aligned. A first click of the pen's button unlocked the cursor, and participants moved it to the opposite target dot while receiving continuous visual feedback. When the cursor was positioned on the target dot, participants pressed the pen's button a second time. Then, both dots as well as the cursor disappeared, and participants started the replication of the hand amplitude by reversing the movement direction with the pen. When they thought to have reproduced the initially performed hand amplitude, they finally pressed the pen's button to terminate the trial. Subsequently, a new trial was presented. Summarizing, trials consisted of two phases each: the initial phase with visual feedback (1) and the inverse replication phase without any visual feedback (2). The non-dominant hand rested relaxed on the participants' lap. The experiment lasted about 30 min.

Experiment 1b consisted of one block of 45 trials with hand amplitudes being 25, 50, or 75 mm (three hand amplitudes with 15 repetitions each, randomly presented). Another six trials were presented in advance to familiarize subjects with the task and procedure (the same three gains as used in the experimental trials with two repetitions each, randomly assigned). In this experiment two experimenters were present: the first experimenter fulfilled the same tasks as described for the experimenter in Experiment 1a, the second experimenter was responsible for presenting the trials (see below).

Before Experiment 1b started, participants were instructed to produce continuous and smooth forth and back movements with the pen without interrupting. They were further instructed to reproduce the initially performed hand amplitude in phase 2 as accurately as possible and to monitor their hand motion in phase 1 carefully. At the beginning of each trial, the second experimenter positioned the start barrier next to the pen and the second barrier at a distance of 25, 50, or 75 mm. A trial started with a first click of the pen's button. Then, participants moved the pen to the opposite barrier, while the second experimenter removed the start barrier. When the pen had reached the opposite barrier, participants pressed the pen's button a second time. They reversed the movement direction and started to reproduce the initially performed hand amplitude. When they thought to have reproduced the initially performed hand amplitude, they finally pressed the pen's button to terminate the trial and the second experimenter presented a new trial. The experiment lasted about 20 min.

Experiment 1a was based on a 2 × 3 design with the within-subject factors *perturbed motion* (cursor motion vs. hand motion) and *length variation* (short vs. middle vs. long). Experiment 1b served as a control experiment with the within-subject factor *hand amplitude* (25 vs. 50 vs. 75 mm). The dependent variable was the mean estimated amplitude (in %), the gain between the observed replicated hand amplitude and the to-be-replicated hand amplitude (= observed replicated hand amplitude / to-be-replicated hand amplitude ^*^ 100). Trials were considered as erroneous and omitted from analyses when the initial movement trajectory was non-continuous (with *v* = 0 within the initial hand movement) and/or its direction changed, when the initial movement overshot the target area, when the second button click occurred while the cursor was outside the target area and when the observed replicated amplitude was shorter than or equal to 10 mm.

#### Participants

For Experiment 1a 17 students (4 female) of the RWTH Aachen University, aged from 18 to 31 years (*M* = 24; *SD* = 4.2) volunteered. All participants were right handed, had normal or corrected-to-normal vision and were naïve with respect to the purpose of the experiment. Another 16 students (9 female) of the RWTH Aachen University, aged from 18 to 36 years (*M* = 24; *SD* = 5.1) volunteered for the control experiment (Experiment 1b). Fourteen of them were right handed, and all of them had normal or corrected-to-normal vision and were naïve with respect to the purpose of the experiment.

### Results

Mean estimated amplitudes (in %) were calculated for error-free trials [error rates at 4.7% (Experiment 1a) and 8.3% (Experiment 1b)]. First, we analyzed data from Experiment 1a using a 2 (*perturbed motion:* cursor motion vs. hand motion) × 3 (*length variation*: short vs. middle vs. long) analysis of variance for repeated measurements (ANOVA). Second, we compared replicated hand amplitudes with and without visual feedback in phase 1 (Experiment 1a, perturbed hand motion vs. Experiment 1b) by using a two-factorial ANOVA for repeated measurements with the within-subject factor hand amplitude (25 vs. 50 vs. 75 mm) and the between subject factor visual feedback (with vs. without visual feedback in phase 1).

**Figure 4:**
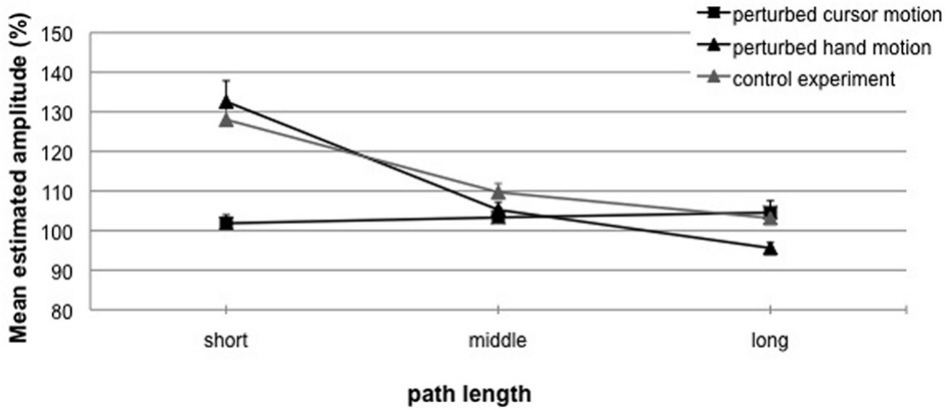
**Experiment 1**. Mean estimated amplitude (%) for perturbed cursor motions (squares) and perturbed hand motions with visual feedback in phase 1 (black triangles), and perturbed hand motions without visual feedback in phase 1 (gray triangles) as a function of length variation. A performance of 100% indicates exact replications. Error bars represent the standard error of the mean.

Figure 4 depicts the results for blocks with perturbed cursor motions (squares) and perturbed hand motions (black triangles). The ANOVA revealed a significant main effect for the factors *perturbed motion* [*F*_(1, 32)_ = 14.35; *p* < 0.01, η^2^ = 0.47] and *length variation* [*F*_(2, 32)_ = 41.70; *p* < 0.01, η^2^ = 0.72], and their significant interaction [*F*_(2, 32)_ = 42.10; *p* < 0.01, η^2^ = 0.73].

For perturbed cursor motions (Figure 4, squares) observed hand amplitudes (phase 2) did not differ from the to-be-replicated hand amplitudes (phase 1: 50 mm). That means for all cursor path lengths (phase 1: 60, 120, or 180 mm) replications were very accurate (*M* = 102% (0.96 mm) vs. 103% (1.77 mm) vs. 106% (2.37 mm); *t*-tests not significant with *p*'s > 0.16). Perturbed hand motions (Figure 4, black triangles) were most accurate when in phase 1 the visual target distance (50 mm) was equal to the performed hand amplitude (50 mm), although observed hand amplitudes deviated from the to-be-replicated hand amplitudes [*M* = 105% (2.60 mm); *t*_(16)_ = 2.66; *p* < 0.05]. When in phase 1 the hand amplitude was short (25 mm), in phase 2 significant overshoots occurred [*M* = 133% (8.17 mm); *t*_(16)_ = 6.23; *p* < 0.01]. When in phase 1 the hand amplitude was long (75 mm), in phase 2 significant undershoots occurred [*M* = 96% (−3.54 mm); *t*_(16)_ = −3.21; *p* < 0.01].

Second, Figure 4 depicts the results for replicated hand amplitudes with (black triangles) and without (gray triangles) visual feedback in phase 1. In Experiment 1b observed hand amplitudes deviated from the to-be-replicated hand amplitudes when the hand amplitude in phase 1 was short or middle [25 mm: *M* = 128% (6.83 mm), *t*_(15)_ = 9.10; *p* < 0.01; 50 mm: 109% (4.92 mm), *t*_(15)_ = 4.42; *p* < 0.01]. Replications were accurate when the hand amplitude in phase 1 was long [75 mm: *M* = 103% (2.76 mm); n.s.]. The ANOVA revealed a significant main effect of the factor hand amplitude [*F*_(2, 62)_ = 101.48; *p* < 0.01; η^2^ = 0.76] and a significant interaction with the factor visual feedback [*F*_(2, 62)_ = 3.93; *p* < 0.05; η^2^ = 0.11]. That means replicated amplitudes without visual feedback in phase 1 were more accurate and did not undershoot when compared with replicated amplitudes with visual feedback in phase 1. Consequently, the increased inaccuracy observed in replicated amplitudes with visual feedback in phase 1 can be interpreted as visual aftereffects.

### Discussion

In this experiment we asked about the impact of dimensional overlap on aftereffects in motor replications. In the condition *perturbed cursor motion* we did not find any aftereffects. That means the variation of cursor path length did not induce aftereffects. Aftereffects occurred in the condition *perturbed hand motion* only. Considering our first hypothesis the results confirm H1b: Aftereffects vary as a function of the relation between hand path length and visual target distance, and not with respect to the relation between hand and cursor path length. Two conclusions can be drawn from these findings: First, the dimensional overlap modulated aftereffects. Concerning the dimension shape, hand motion (phases 1 and 2) and visual target distance (phase 1) did overlap, but hand motion (phases 1 and 2) and cursor motion (phase 1) did not overlap. Consequently, visual aftereffects appeared from length variations between hand path length and visual target distance, but not from cursor path length variations. However, the restrictions of measuring deviations along the x-axis only will be discussed later in more detail.

Second, manual actions are pre-programmed on the basis of target amplitude and target width (Fitts' law; Fitts, 1954). In other words, they are cognitively represented with respect to the action's goal (James, 1890; Greenwald, 1970; Hommel et al., 2001). In our task (phase 1), target amplitude was the distance between start dot and target dot. Thus, our finding also supports the notion of the ideo-motor principle and action effect account (James, 1890; Greenwald, 1970; Hommel et al., 2001). The present experimental setting, more generally speaking every visual target presentation, does not allow distinguishing between both conclusions. This point will also be discussed later.

Furthermore, aftereffects in the condition *perturbed hand motion* (8, 3, and –4 mm; range 12 mm) are considerably smaller than that obtained in a similar condition by Ladwig et al.. (2012; Figure 3, asterisks: 24, 7, and –8 mm; range 32 mm). There is a simple explanation for this. Hand path length and visual target distance in the present experiment is 50 mm (1:1 condition) and therefore shorter compared to the 120 mm amplitude used by Ladwig et al. (2012). If one accounts for the amplitudes the ratio between the range of aftereffects and the hand amplitude remains nearly the same (12/50 and 32/120 with ratios being 0.24 and 0.27, respectively). Consequently, it makes sense that we find in the present experiment smaller aftereffects for the smaller hand amplitudes.

## Experiment 2

### Methods

#### Stimuli, design, and procedure

These were the same as in Experiment 1, except for the constancy of either cursor or hand motion and set size of presented trials per block. Instead of the blocked presentation of trials with perturbed cursor motion (one block, set size: 3) or hand motion (another block, set size: 3), we presented trials with perturbed cursor motions and perturbed hand motions randomly within a block (set size: 5) to increase contextual interference. The two blocks consisted of 48 trials each (the same 2 × 3 combinations of perturbed motion and length variation as in Experiment 1 with 8 repetitions each, randomly presented) and another six trials presented in advance of each block in order to familiarize subjects with the task (the same 2 × 3 combinations of perturbed motion and length variation as used in the experimental trials with one repetition each, randomly assigned). The experiment lasted 30 min.

#### Participants

Another 14 students (5 female) of the RWTH Aachen University, aged from 17 to 34 years (*M* = 26; *SD* = 4.5) volunteered for the experiment. All but one participant were right handed. All participants had normal or corrected-to-normal vision and were naïve with respect to the purpose of the experiment.

### Results

Again, mean estimated amplitudes (in %) were calculated for error-free trials (error rate at 11.6%) and analyzed using a 2 (*perturbed motion:* cursor motion vs. hand motion) × 3 (*length variation*: short vs. middle vs. long) ANOVA for repeated measurements. Additionally, we compared replicated hand amplitudes with and without visual feedback in phase 1 (Experiment 2, perturbed hand motion vs. Experiment 1b) by using a two-factorial ANOVA for repeated measurements with the within-subject factor hand amplitude (25 vs. 50 vs. 75 mm) and the between subject factor visual feedback (with vs. without visual feedback in phase 1).

**Figure 5:**
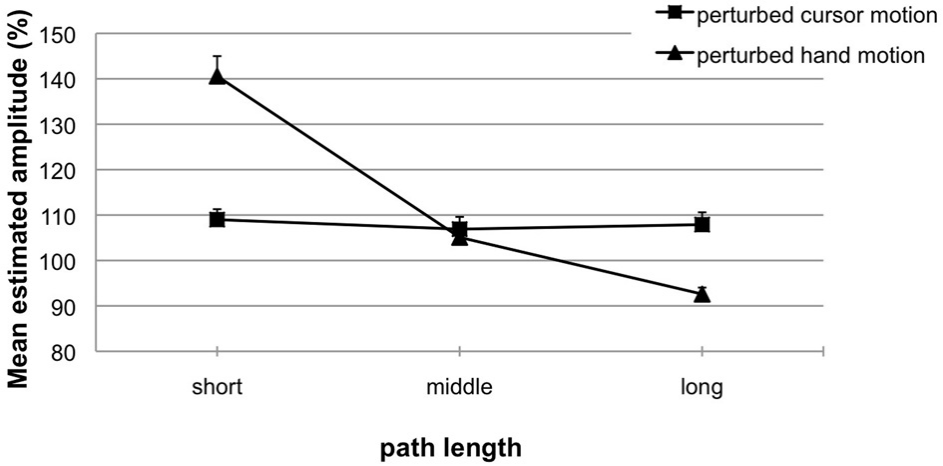
**Experiment 2**. Mean estimated amplitude (%) for perturbed cursor motions (squares) and perturbed hand motions (triangles) as a function of length variation. A performance of 100% indicates exact replications. Error bars represent the standard error of the mean.

Figure 5 depicts the results for blocks with perturbed cursor motions (squares) and perturbed hand motions (triangles). The ANOVA revealed significant main effects for the factors *perturbed motion* [*F*_(1, 13)_ = 20.42; *p* < 0.01, η^2^ = 0.61] and *length variation* [*F*_(2, 26)_ = 33.02; *p* < 0.01, η^2^ = 0.71], and their significant interaction [*F*_(2, 26)_ = 32.18; *p* < 0.01, η^2^ = 0.71].

For perturbed cursor motions (Figure 5, squares) observed hand amplitudes (phase 2) were quite accurate and did not differ from the to-be-replicated hand amplitudes (phase 1: 50 mm). That means the variation of cursor path length (phase 1: 60, 120, or 180 mm) did not induce any aftereffects [*M* = 109% (4.40 mm) vs. 107% (3.56 mm) vs. 108% (4.08 mm); *t*-tests not significant with *p*'s > 0.058]. Perturbed hand motions (Figure 5, triangles) were very accurately replicated when the to-be-replicated hand amplitude (phase 1) was 50 mm [*M* = 105% (2.66 mm); n.s.]. When in phase 1 the to-be-replicated hand amplitude was short (25 mm), significant overshoots occurred in phase 2 [*M* = 141% (10.47 mm); *t*_(13)_ = 4.95; *p* < 0.01]. When in phase 1 the to-be-replicated hand amplitude was long (75 mm), significant undershoots occurred in phase 2 [*M* = 93% (−5.74 mm); *t*_(13)_ = −2.75; *p* < 0.05]. Again, we compared replicated hand amplitudes with and without visual feedback in phase 1 (Experiment 2, perturbed hand motion vs. Experiment 1b). The ANOVA revealed a significant main effect of the factor hand amplitude [*F*_(2, 56)_ = 83.71; *p* < 0.01; η^2^ = 0.74] and a significant interaction with the factor visual feedback [*F*_(2, 56)_ = 8.47; *p* < 0.01; η^2^ = 0.23]. That means replicated amplitudes without visual feedback in phase 1 were more accurate and did not undershoot when compared with replicated amplitudes with visual feedback in phase 1. Again, this proofs that the increased inaccuracy observed in replicated amplitudes with visual feedback in phase 1 are visual aftereffects.

Further analyses were done to investigate the impact of contextual interference (lower in Experiment 1 than in Experiment 2) on aftereffects in motor replications. For the conditions perturbed cursor motions and perturbed hand motions estimated amplitudes (%) were analyzed separately using a 2 [*contextual interference:* low (Experiment 1) vs. high (Experiment 2)] × 3 (*length variation*: short vs. middle vs. long) mixed ANOVA for repeated measurements. For perturbed cursor motions the analysis did not reveal any significant main effect or interaction (all *p*'s > 0.28). For perturbed hand motions the ANOVA confirmed the significant main effect of the factor *length variation* [*F*_(2, 58)_ = 87.74; *p* < 0.01, η^2^ = 0.75]. Other effects did not reach significance (*p* > 0.24).

**Figure 6:**
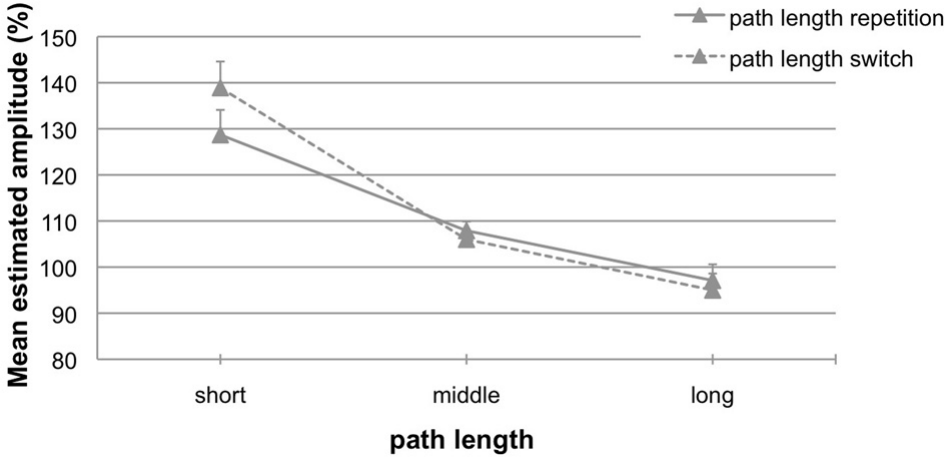
**Mean estimated amplitude (%) for trials with path length repetitions (solid line) and path length switches (dashed line) in the condition *perturbed hand motions***. A performance of 100% indicates exact replications. Error bars represent the standard error of the mean.

To address our research question of how experience shapes subsequent motor behavior we comprised data from both experiments, since aftereffects did not differ between them. Data were analyzed separately for the conditions *perturbed cursor motion* and *perturbed hand motion*. For perturbed cursor motions (24% repetition trials, 76% switch trials) the 2 (*path length switch*: repetition vs. switch) × 3 (*length variation*) ANOVA for repeated measurements did not reveal any significant main effects or interaction (all *p*'s > 0.27). For perturbed hand motions (27% repetition trials, 73% switch trials) the ANOVA confirmed the significant main effect of the factor *length variation* [*F*_(2, 50)_ = 53.92; *p* < 0.01, η^2^ = 0.68] and a significant interaction between *length variation* and *path length switch* [*F*_(2, 50)_ = 10.31; *p* < 0.01, η^2^ = 0.29]. The main effect *path length switch* was not significant (*p* = 0.15). Figure 6 depicts the results for the condition with perturbed hand motions. There are switch costs for path length changes that result in larger aftereffects [Figure 6, dashed line; 139% (10.0 mm), 106% (3.1 mm), and 95% (−3.8 mm); range 44% (13.8 mm)] compared to path length repetitions [Figure 6, solid line; 129% (7.4 mm), 108% (4.0 mm), and 97% (−2.2 mm); range 32% (9.6 mm)].

### Discussion

Again, we did not find any aftereffects in the condition *perturbed cursor motion*. But in the condition *perturbed hand motion* replications in phase 2 varied as a function of visual target distance. The finding replicates the pattern of results from Experiment 1, and supports hypothesis 1b once more.

In our second hypothesis we assumed that the motion constancy and the smaller set size in Experiment 1 would benefit motor replications in phase 2, and predicted smaller aftereffects in Experiment 1 than in Experiment 2. Although the data show a numerical increase in aftereffects the way we predicted, differences were not statistically significant. We will open a deeper discussion on that in the following section.

Finally, data exploration concerning a performance benefit in gain repetitions revealed a significant reduction of aftereffects for repetitions compared to switches. The pattern of results is similar to that found by Verstynen and Sabes (2011), who demonstrated the adaptation benefit for angular deviations. However, the task of the present experiment did not address adaptation. Remember, participants did not receive any visual feedback in phase 2, where they had to replicate the hand amplitude from phase 1 as accurately as possible. It is known that movements are usually pre-programmed with the previously-used internal model (Wolpert and Flanagan, 2001). Deviations between predicted and actual outcome reflect the specifications of the pre-programmed movement, and are usually corrected online when they become apparent. In this way, the forward model is continuously adjusted. We assumed that gain repetitions, and more specifically the closed-loop control in phase 1 functioned in a way to adjust the internal model. This seemed to be the case and smaller aftereffects occurred for gain repetitions than for gain switches.

## General discussion

The aim of the present study was to investigate the contribution of dimensional overlap on aftereffects in motor replications. The task, adapted from Ladwig et al. (2012) was to move a cursor on the display from a start position to a target. The cursor motion followed the shape of an inverted U while the hand motion followed a straight horizontal line (phase 1). Then movement direction had to be inverted to replicate the formerly performed hand amplitude on the return without visual feedback (phase 2). The variation of length and shape of hand and cursor motion in phase 1 decoupled two different relations in visually controlled aiming movements (Table 1): First, the relation between hand motion and cursor motion (dissimilar length and dissimilar shape = dimensions did not overlap), and second, the relation between hand motion and visual target distance [similar or dissimilar length (dimension did or did not overlap) and similar shape (dimensional overlap)].

Both experiments confirm that aftereffects occur when dimensions between visual and proprioceptive action effects overlap. Thus, when the shape of hand motion and visual target distance was similar, and the hand path length (in phase 1) was shorter (longer) than the visual target distance, participants overshot (undershot) in phase 2. This pattern of aftereffects in terms of systematic over- and undershoots was observed in several studies by Ladwig et al. (2012, 2013) for motor responses. In their experiments, even though the hand amplitude was constant, the varying cursor amplitude in phase 1 produced aftereffects in phase 2. In this condition movements could have been performed without any corrections of the previously-used motor program (Wolpert and Flanagan, 2001), but they weren't. That motor replications were still influenced by the formerly perceived visual information speaks in favor of a common representational domain for sensory information belonging to the same event (e.g., Prinz, 1997; Hommel et al., 2001). The common representation in form of an event code makes it possible for sensory information to interact with each other and to influence subsequent actions. However, this is not the whole explanation of the present findings.

Comparing conditions with and without visual feedback in phase 1 should clarify the impact of visual feedback on observed deviations. We assumed that if visual feedback in phase 1 induced deviations in phase 2, then the hypothesized pattern of over- and undershoots should occur in Experiment 1a and 2, but not in Experiment 1b. But this was not what we found. Deviations in phase 2 were present in both conditions, and although deviations were considerably smaller without visual feedback, they were in the same direction as compared to the condition with visual feedback. This strongly points at other factors—besides visual feedback—that influence motor replications in phase 2, for instance a regression-to-the-mean effect (Teghtsoonian and Teghtsoonian, 1978). In our experiments the middle path length (50 mm) represents the mean length. In the condition without visual feedback motor replications of the short (25 mm) and the long path (75 mm) length deviated about nearly the same amount from the “mean” (short-middle: Δ 1.91 mm; long-middle: Δ 2.16 mm). And, the larger deviations in the condition with visual feedback showed the same symmetry around the mean.

Rieger et al. (2005) found the same pattern of over- and undershoots when investigating the compensation for gain changes. Participants were asked to perform up- and downward strokes between two visual target lines by moving a pen on a covered digitizer tablet up and down. After six baseline strokes (gain 1:1) a gain was introduced for further six strokes. In one experimental condition the gain resulted in constant cursor amplitudes while the hand amplitude was shorter or longer (cf. Ladwig et al., 2012: condition varying hand amplitude). In another experimental condition the gain resulted in constant hand amplitudes while the cursor amplitude was shorter or longer (cf. Ladwig et al., 2012: condition constant hand amplitude). After that, another six baseline strokes were presented. Compensation for changes was measured by analyzing the deviation from the target line (in mm) for the first stroke performed after an experimental condition. When the hand amplitude (both experimental conditions) was longer (shorter) than the cursor amplitude, undershoots (overshoots) occurred in the first stroke performed afterwards. This result closely resembles the pattern found by Ladwig et al. (2012, 2013) as well as the pattern found in the present experiment for perturbed hand amplitudes. Although the differences between experimental tasks don't allow a direct comparison, the finding—that subsequent motor actions are influenced by formerly perceived visual information—is again in line with the predictions of common coding approaches (e.g., Prinz, 1997; Hommel et al., 2001).

Considering the impact of dimensional overlap on motor replications further, Ladwig et al. (2012) reduced the overlap between visual and proprioceptive action effects along one dimension. In one condition (Ladwig et al., 2012; Experiment 1) a 90° rotation of the visual cursor motion resulted in upward-downward movements of the cursor when the hand produced horizontal leftward-rightward movements on the tablet. Consequently, the orientation of hand and cursor motion did no longer overlap (horizontal vs. vertical). The shape was still similar (linear movements = dimensional overlap) and the length was either similar or not (dimension did or did not overlap). Aftereffects in terms of over- and undershoots were still significantly present. But they were considerably smaller when the dimensional overlap was limited (horizontal vs. vertical) compared to when dimensions did overlap (both motions horizontal). In the present experiments dimensional overlap concerns the shape (linear vs. curved) and length of motion (similar vs. dissimilar). Concerning shape, hand motion and visual target distance did overlap (both linear), but hand motion and cursor motion did not overlap (linear vs. curved). We observed aftereffects depending on length variations between hand path length and visual target distance only. However, future studies should also consider measuring deviations along the y-axis as well. We did not observe any aftereffects from curved amplitudes along the x-axis. But, if hand movements were not restricted along the y-axis as in our experiments, aftereffects from the curved amplitude could have been observed. Measuring deviations along both axes allow distinguishing between “length-aftereffects” (= deviations along the x-axis) and “shape-aftereffects” (= deviations along the y-axis).

Further experiments are necessary to fully confirm our conclusion about the dimensional overlap being responsible for aftereffects. If it is the dimensional overlap between visual and proprioceptive effects and not (only) the cognitive representation of the action's goal (James, 1890; Greenwald, 1970; Hommel et al., 2001) then performing curved hand motions instead of linear ones should lead to the pattern of aftereffects we predicted in hypothesis 1a.

To sum up, although in visually controlled manual movements visual and proprioceptive action effects might not be integrated—for instance because they do not overlap or are spatially separated (e.g., Ernst and Banks, 2002; Gepshtein et al., 2005)—they nevertheless affect motor performance in terms of aftereffects.

The data could not support our second hypothesis, in which we expected smaller aftereffects in Experiment 1 than in Experiment 2, because of the simplified context in phase 1. In Experiment 1 always one aspect of the task in phase 1 remained constant within a block, either the hand motion or the cursor motion. This constancy and the smaller set size of contexts to be learned should benefit motor replications in phase 2. Aftereffects numerically increased the way we predicted, however, differences were not significant. There are several speculations why this happened. First, in both experiments trials randomly varied, and although motion constancy and set size differed it seems that the contextual changes between Experiments 1 and 2 were not very distinct and did not induce (enough) interference. Second, in our experiments, the mapping between hand path length and visual action effects was very simple (short vs. middle vs. long) and consisted of 5 different trials in total. Participants could have been able to acquire implicit knowledge about the transformations. A higher number of gain factors should increase contextual interference. In a yet unpublished experiment a signal between phases 1 and 2 indicated whether participants had to reproduce the hand motion or the cursor motion in phase 2. Aftereffects considerably increased when compared to a blocked reproduction of either hand or cursor amplitude. This could be another manipulation to increase contextual interference.

The data exploration of gain repetition and gain switch-trials supports the view that gain repetitions adjusted the forward model. It seemed to become more accurate so that smaller aftereffects occurred in gain repetitions than in gain switches (range of aftereffects: 9.6 mm vs. 13.8 mm). Aftereffects significantly dropped by 4.2 mm; that is a 30% benefit from a repeated prior trial. In the present experiment we did not control for the number of gain repetitions and gain switches. Comparable to former studies in our lab (e.g., Ladwig et al., 2012, 2013) trials were presented completely randomly to control for confounds in task presentation. Nevertheless, the results are quite promising, and further experiments will give a more detailed insight into the processes of sensorimotor control. Finally, one could assume that aftereffects are not (much) influenced by linear mappings, but might be more affected by dynamic mappings. For the latter action effects become less predictive, and research on these kinds of transformations demonstrate great inaccuracies in motor behavior. Moreover, users are not able to fully acquire a correct cognitive representation of the transformations, but approximate the internal model (e.g., Sülzenbrück and Heuer, 2009).

In conclusion, dimensional overlap between visual and proprioceptive action effects modulates human information processing in visually controlled actions. However, adjustment of the internal model seems to occur very fast for this kind of simple linear transformation, so that the impact of prior visual feedback is fleeting.

### Conflict of interest statement

The authors declare that the research was conducted in the absence of any commercial or financial relationships that could be construed as a potential conflict of interest.
